# Genome Sequence of the Deep-Sea Bacterium *Idiomarina abyssalis* KMM 227^T^

**DOI:** 10.1128/genomeA.01256-15

**Published:** 2015-10-29

**Authors:** Bruce A. Rheaume, Scott Mithoefer, Kyle S. MacLea

**Affiliations:** a Biology Program, University of New Hampshire, Manchester, New Hampshire, USA; b Biotechnology Program, University of New Hampshire, Manchester, New Hampshire, USA

## Abstract

*Idiomarina abyssalis* KMM 227^T^ is an aerobic flagellar gammaproteobacterium found at a depth of 4,000 to 5,000 m below sea level in the Pacific Ocean. This paper presents a draft genome sequence for *I. abyssalis* KMM 227^T^, with a predicted composition of 2,684,812 bp (47.15% G+C content) and 2,611 genes, of which 2,508 were predicted coding sequences.

## GENOME ANNOUNCEMENT

*Idiomarina abyssalis* strain KMM 227^T^, the first member of its genus to be described, is a Gram-negative, flagellar, strictly aerobic gammaproteobacterium isolated from seawater taken in 1985 from 4,000 to 5,000 m below the surface of the northwestern Pacific Ocean (1). Since its characterization in 2000, *Idiomarina* has grown to accommodate several other species, many of which have published genomes, including *I. xiamenensis* (2), *Idiomarina* sp. strain A28L (3), and *Idiomarina* sp. strain 28-8 (4); all of these species were found in saline environments or marine sediment.

*I. abyssalis* is an obligate halophile that does not utilize glucose, fructose, mannitol, sucrose, maltose, or lactose (1); however, similar to other species of *Idiomarina, I. abyssalis* appears to utilize amino acids for carbon and energy (1–6). *I. abyssalis* contains predominantly iso-branched fatty acids, typically with 15 or 17 carbons (1). It grows under varied conditions of temperature, pH, and salinity, and it was isolated from depths at up to ~500 atmospheres (atm) pressure. It has been shown that hydrostatic pressure of this magnitude can “sterilize” many species of bacteria (7); it is unclear, however, if *I. abyssalis* is active under these extreme pressures or if the organism is existing in a viable but nonculturable (VBNC) state (8).

*I. abyssalis* KMM 227^T^ was obtained from ATCC (BAA-312) in freeze-dried form. *I. abyssalis* was rehydrated and cultured in marine broth and incubated at 30°C for 72 h at atmospheric pressure. After rehydration*,* the bacterium was cultured in log-phase growth before its genomic DNA (gDNA) was obtained. Extraction of gDNA was performed using the Genomic-tip 500/g kit (Qiagen, Valencia, CA). The gDNA was fragmented and tagged with adapters using a Nextera library prep kit, and the library of sequences was analyzed using an Illumina HiSeq 2500 sequencer to generate 150-bp paired-end reads at the Hubbard Center for Genome Studies. Bioinformatic removal of adapter sequences and trimming were performed prior to gene finding, annotation, and analyses using Trimmomatic (9).

The *I. abyssalis* genome sequencing resulted in a total of 1,010,746 reads with an average length of 147,475 bp. These reads were assembled into 68 contigs using SPAdes version 3.5.0 (10). The contigs were analyzed using QUAST version 2.3 and were found to have a total length of 2,684,812 bp and an average coverage of 13.2× (11). The largest contig was 983,913 bp, with an *N*_50_ value of 170,438 bp and a G+C content of 47.15%, just under the figure of 50% reported by Ivanova et al. (1). The National Center for Bioinformatics (NCBI) automatic annotation pipeline was used for genome annotation (12). A total of 2,611 genes, 2,508 coding sequences (CDSs), 40 pseudogenes, seven rRNAs, 55 tRNAs, and one noncoding RNA (ncRNA) were discovered using the NCBI pipeline. Comparisons of this genome with the *Idiomarina zobellii* genome and others will enable a more comprehensive metabolic and genetic study of adaptations to different saline environments.

### Nucleotide sequence accession numbers. 

This whole-genome shotgun project has been deposited at DDBJ/EMBL/GenBank under the accession no. LGOW00000000. The version described in this paper is version LGOW01000000.
